# Germinal *GLT8D1*, *GATAD2A* and *SLC25A39* mutations in a patient with a glomangiopericytal tumor and five different sarcomas over a 10-year period

**DOI:** 10.1038/s41598-021-88671-0

**Published:** 2021-05-07

**Authors:** Arnaud Beddok, Gaëlle Pérot, Sophie Le Guellec, Noémie Thebault, Alexandre Coutte, Henri Sevestre, Bruno Chauffert, Frédéric Chibon

**Affiliations:** 1 Radiation Oncology Department, Curie Institute, 25 rue d’Ulm, 75005 Paris, France; 2 INSERM U1037, Cancer Research Center in Toulouse (CRCT), Toulouse, France; 3 CHU de Toulouse, IUCT-Oncopole, Toulouse, France; 4 Department of Pathology, Institut Claudius Régaud, IUCT-Oncopole, Toulouse, France; 5 Radiation Oncology Department, CHU Amiens, Picardie, France; 6 Pathology Department, CHU Amiens, Picardie, France; 7 Oncology Department, CHU Amiens, Picardie, France

**Keywords:** Cancer, Cancer genetics, Sarcoma

## Abstract

Soft tissue sarcoma represents about 1% of all adult cancers. Occurrence of multiple sarcomas in a same individual cannot be fortuitous. A 72-year-old patient had between 2007 and 2016 a glomangiopericytal tumor of the right forearm and a succession of sarcomas of the extremities: a leiomyosarcoma of the left buttock, a myxofibrosarcoma (MFS) of the right forearm, a MFS of the left scapula, a left latero-thoracic MFS and two undifferentiated sarcomas on the left forearm. Pathological examination of the six locations was not in favor of disease with local/distant recurrences but could not confirm different diseases. An extensive molecular analysis including DNA-array, RNA-sequencing and DNA-Sanger-sequencing, was thus performed to determine the link between them. The genomic profile of the glomangiopericytal tumor and the six sarcomas revealed that five sarcomas were different diseases and one was the local recurrence of the glomangiopericytal tumor. While the chromosomal alterations in the six tumors were different, a common somatic *CDKN2A/CDKN2B* deletion was identified. RNA-sequencing of five tumors identified mutations in *GLT8D1*, *GATAD2A* and *SLC25A39* in all samples. The germline origin of these mutations was confirmed by Sanger-sequencing. Innovative molecular analysis methods have made possible a better understanding of the complex tumorigenesis of multiple sarcomas.

## Introduction

Soft tissue sarcomas (STS) are rare (1% of all cancers, 3.6 per 100,000)^1^. The STS classification is historically based on histological subtypes and grading^2^. However, there is a wide variety of different histological subtypes of sarcomas and they may be difficult to differentiate^3^. In recent decades, advanced molecular techniques and genetic profiling have revolutionized the approach to sarcoma classification^4^. One third of all STS are characterized by recurrent specific chromosomal translocations, resulting in fusions of specific genes, usually encoding aberrant chimeric transcription factors. The other two-thirds of STS have no genetic signature and are characterized by numerous aberrations, including chromosomal losses and gains^5^. These sarcomas are more frequent and are generally high-grade, including undifferentiated pleomorphic sarcomas (UPS) and leiomyosarcomas (LMS). This group of tumors has a high prevalence of p53 control point alterations, including *TP53* inactivating mutations and homozygous deletion of *CDKN2A*^6^. Several genetic syndromes are associated with an increased risk of STS, particularly Li-Fraumeni syndrome^7^. In the present study, we report the unique case of a 72-year-old patient who presented with glomangiopericytal tumor followed by six sarcomas of the extremities between 2007 and 2016. Pathological examination of the tumors could not separate a unique disease with local/distant recurrences or a succession of different diseases. An extensive molecular and genetic analysis was then performed to determine the tumorigenesis in this patient.

## Material and methods

### Patient and samples

**Figure 1:**
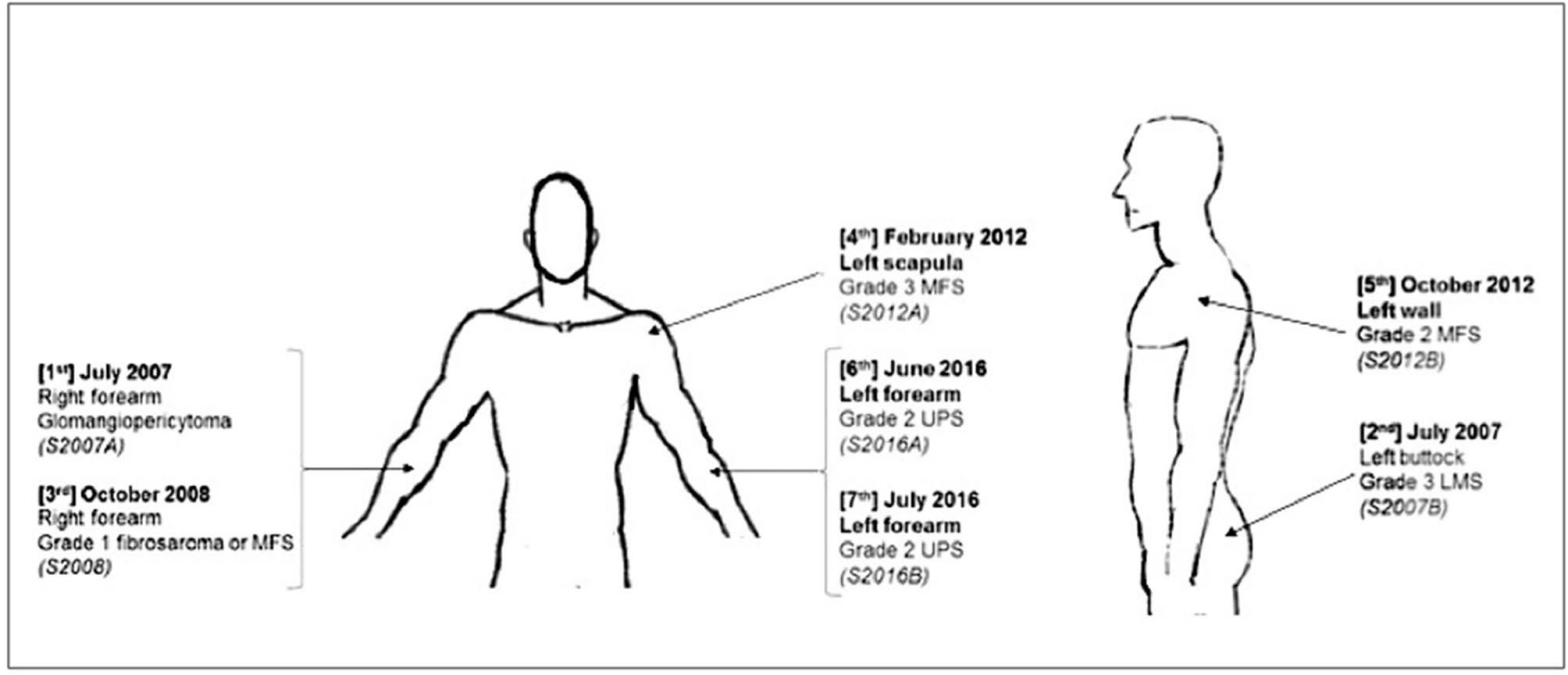
Location and histological subtypes of the seven soft tissue tumors. *LMS* leiomyosarcoma, *MFS* myxofibrosarcoma, *UPS* undifferentiated pleomorphic sarcoma.

**Figure 2:**
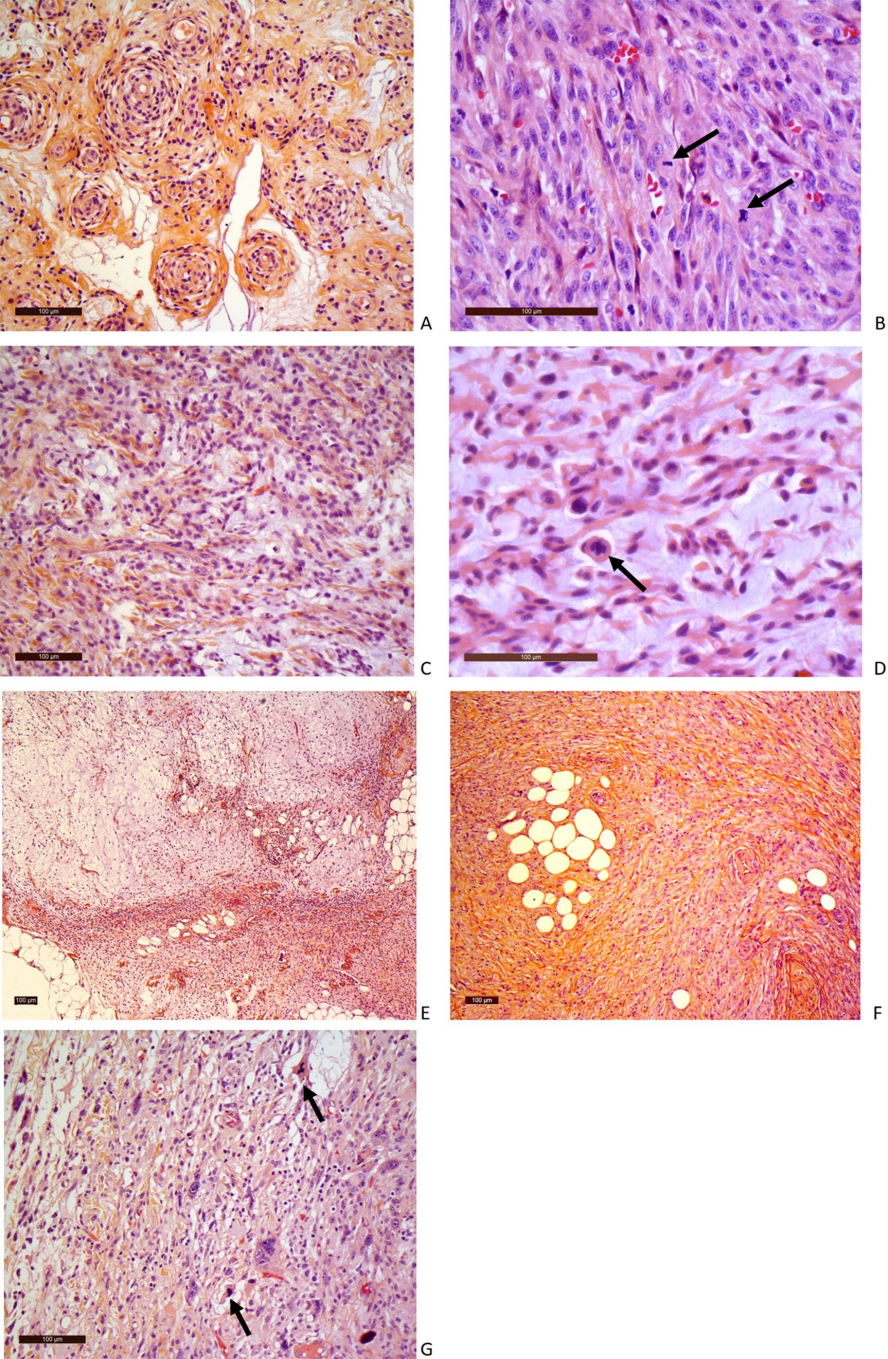
Histological characteristics of the soft tissue tumors. **(A)** S2007A: glomangiopericytal tumor with uncertain potential: ovoid cells embedded in a myxoid background and concentrically arranged around rounded vascular structures. The tumor is classified according to the WHO classification as a tumor with uncertain potential because of its size. **(B)** S2007B: grade 3 LMS: proliferation consisting of long bundles composed of spindle cells with abundant eosinophilic cytoplasm. Black arrow: atypical mitosis. **(C)** S2008: Low grade MFS: slightly atypical fusiform cells embedded in myxoid background. Numerous thin-walled vessels. **(D)** S2012A: MFS: High Power magnification of atypical spindle cells. Black arrow: atypical mitosis. **(E)** S2012B: MFS: At low magnification power, juxtaposition of dense spindle cell area and paucicellular myxoid proliferation. **(F)** S2016A: grade 2 UPS: proliferation of spindle cells dissecting adipose tissue (at the bottom). **(G)** S2016B: grade 2 UPS: atypical spindle and pleomorphic cells on a myxoid background. Black arrow: atypical mitosis. *LMS* leiomyosarcoma, *HE* hematoxylin and eosin staining, *MFS* myxofibrosarcoma, *UPS* undifferentiated pleomorphic sarcoma. Scale bar: 100 µm.

Molecular analysis was based on the case of a 72-year-old patient with a history of ear melanoma and pulmonary sarcoidosis with mediastinal lymph nodes, who presented a glomangiopericytal tumor and a succession of six sarcomas of the extremities (Figs. 1, 2 and Table S1). In July 2007, he synchronously developed a tumor of the right forearm and a tumor of the left buttock. The former was surgically removed and proved to be a glomangiopericytal tumor of uncertain potential (named in our study S2007A). The tumor of the left buttock was widely excised and the diagnosis was a grade 3 (FNCLCC Grading System) LMS (S2007B). In October 2008, he presented with a slowly enlarging mass on the right forearm. Histological analysis revealed features of fibroblastic differentiation with spindle-shaped cells containing elongated nuclei within a myxoid matrix. The diagnosis was a grade 1 fibrosarcoma or myxofibrosarcoma (MFS, S2008). In February 2012, he presented with a subcutaneous tumor on the left scapula. Histological analysis revealed spindle-shaped cells with high mitotic activity, darkly staining nuclei with variably prominent nucleoli and eosinophilic cytoplasm. The stroma contained variable collagen with myxoid and fibrotic areas. The diagnosis of grade 3 MFS was made (S2012A). In October 2012, he presented with an enlarging left latero-thoracic sub-cutaneous mass that was considered a superficial grade 2 MFS (S2012B). In April 2016, he presented a mass on the left forearm. Histological analysis revealed pleomorphic spindle-shaped cells with high mitotic activity. The diagnosis of grade 2 UPS was made (S2016A). In June 2016, he presented a grade 2 UPS in the left forearm (S2016B). The patient finally died in June 2018 from diffuse metastatic evolution (including bone and lung metastases) that did not respond to chemotherapy. No material was available for the melanoma and for the bone and lung metastases. Before performing the analyses, the patient provided written informed consent for use of all specimens for the purpose of the study. All cases have been reviewed by an expert pathologist (SLG) of the French Sarcoma Group according to the World Health Organization^3^. All experimental protocols were approved by the ethical committee of the University Hospital Center of Amiens. All methods were carried out in accordance with relevant guidelines and regulations.

### DNA extraction

Areas of high tumor cellularity (> 80%) or healthy tissue were defined by a pathologist on a hematoxylin and eosin-stained histologic section and were transferred to a formalin-fixed paraffin-embedded (FFPE) tumor block. DNA was extracted using the QIAamp DSP DNA FFPE Tissue Kit (Qiagen, Hilden, Germany) according to the manufacturer’s recommendations. Genomic DNA was then quantified using a Nanodrop 1000 spectrophotometer (Thermo Fisher Scientific, Waltham, MA, USA) and a Qubit fluorometer (Thermo Fisher Scientific, Waltham, MA, USA) using the Qubit dsDNA BR Assay Kit according to the manufacturer’s instructions (Thermo Fisher Scientific, Waltham, MA, USA).

### DNA array

Genomic profiling was performed using the Affymetrix OncoScan CNV Arrays (Thermo Fisher Scientific, Waltham, MA, USA) according to the manufacturer’s instructions. Profiles were visualized and analyzed with the Chromosome Analysis Suite Software (Thermo Fisher Scientific, Waltham, MA, USA) and the annotations of the genome version GRCH37 (hg19). Ploidy was evaluated by analyzing allele difference, according to the manufacturer’s instructions (P/N CL00731).

### RNA extraction

RNA extraction and quality assessment of FFPE tissue were performed as described in Lesluyes et al.^8^.

### RNA sequencing

The RNA sequencing libraries from FFPE tissue sample total RNA were prepared at the Centre Nacional d'Anàlisi Genòmica (CNAG, Barcelona, Spain) using a modified TruSeq RNA Sample Prep Kit v2 protocol (Illumina, Inc., San Diego, CA, USA) as previously described in Lesluyes et al.^8^. Each library was sequenced using TruSeq SBS Kit v3-HS in paired end mode with the read length 2 × 76 bp on HiSeq2000 (Illumina, Inc., San Diego, CA, USA) following the manufacturer’s protocol, as described in Lesluyes et al.^8^.

### Bioinformatics analysis pipeline for RNA sequencing

RNA sequences were aligned using STAR v2.6.0c^9^ with default parameters on the Human Genome version hg38. Thus, duplicated PCR reads were removed with PicardTools v2.18.2118 (http://broadinstitute.github.io/picard/index.html). SNV were detected by BCFtools mpileup v1.6 with a minimum 20 of phred quality (-Q 20) and by BCFtools call-Ac^10^. Thus, variants with fewer than 5 total reads and 2 alternative reads were filtered out. Finally, they were annotated with Annovar v20160201 tool^11^.

### Filtering of RNA-seq data

After the RNA-seq pipeline treatment, 650,497 variants were detected. First, we kept only the exonic and splicing mutations that were not synonymous or not identified in dbSNP or in the 1000 Genomes databases, *i.e*. 4507 mutations. Second, to remove mutations potentially due to fixation artefacts, mutations also found in at least 8/17 of the 17 FFPE samples from a previously published cohort^8^ were removed after conversion of coordinates with LiftOver (hg19 to hg38; https://genome.ucsc.edu/cgi-bin/hgLiftOver). Thus, only 6 mutations were removed. Finally, only exonic mutations detected in all the five studied tumors were kept for further analysis, i.e. 77 exonic mutations (Tables S2 and S3). All mutations were then visualized in the five samples (S2007A, S2007B, S2008, S2012A and S2016A) using the Integrative Genomics Viewer (IGV_2.6.3)^12^. The Ensemble Genome Browser was consulted to ensure that the mutations were not referenced in the dbSNP database and alternative reads were aligned on the genome in some cases using the blat function on the UCSC website. Thus 53/77 (68.8%) variations were removed since they were found in dbSNP, 7/77 (9.1%) mutations were excluded because of a wrong alignment or multiple alignments in other genes, 2/77 (2.6%) mutations were not considered due to a sequencing error and 9/77 (11.7%) mutations were excluded because of a low percentage of alternative reads in most cases. Therefore, only 6 mutations were kept for further validation (7.8%) (Tables S2 and S3).

### Fusion transcript analysis

DeFuse (v0.6.2) was used with ENSEMBL GRCh37.74 annotations and candidate fusions were filtered as previously described^13^.

### PCR on genomic DNA and Sanger sequencing

To analyze the sequences of *p14*^*ARF*^ and *p16*^*INK4A*^, we used the primers described by Iwato et al. for exons 1 and the common exon 2^14^. For common exon 3 and CDKN2A IVS2-105A/G, we used the primers described by Laud et al.^15^. PCR primers were designed using the Primer 3 program (http://frodo.wi.mit.edu/primer3/) for *CDKN2B* mutation screening and for validation of *GLT8D1*, *GATAD2A*, *SLC25A39*, *AZIN1*, *COG3* and *COPA* mutations on genomic DNA (see Table S4). Regarding *CDKN2A* and *CDKN2B* screening, all exons were sequenced in HT2013 and Sarc2011 and only those of *CDKN2A* in HT2012 (insufficient material to sequence all exons of both genes). All PCR were performed on 50 ng of gDNA using AmpliTaqGold DNA polymerase (Thermo Fisher Scientific, Waltham, MA, USA) according to the manufacturer’s instructions and using the following Touch-down PCR program: 2 cycles at 60 °C, followed by 2 cycles at 59 °C, 2 cycles at 58 °C, 3 cycles at 57 °C, 3 cycles at 56 °C, 4 cycles at 55 °C, 4 cycles at 54 °C, 5 cycles at 53 °C and finally 10 cycles at 52 °C. Sanger sequencing was performed by Genoscreen (Lille, France). Sequence electrophoregrams were obtained using FinchTV software (version 1.4.0, Geospiza Inc., Seattle, WA, USA).

## Results

### Tumor genomic profiles

**Figure 3:**
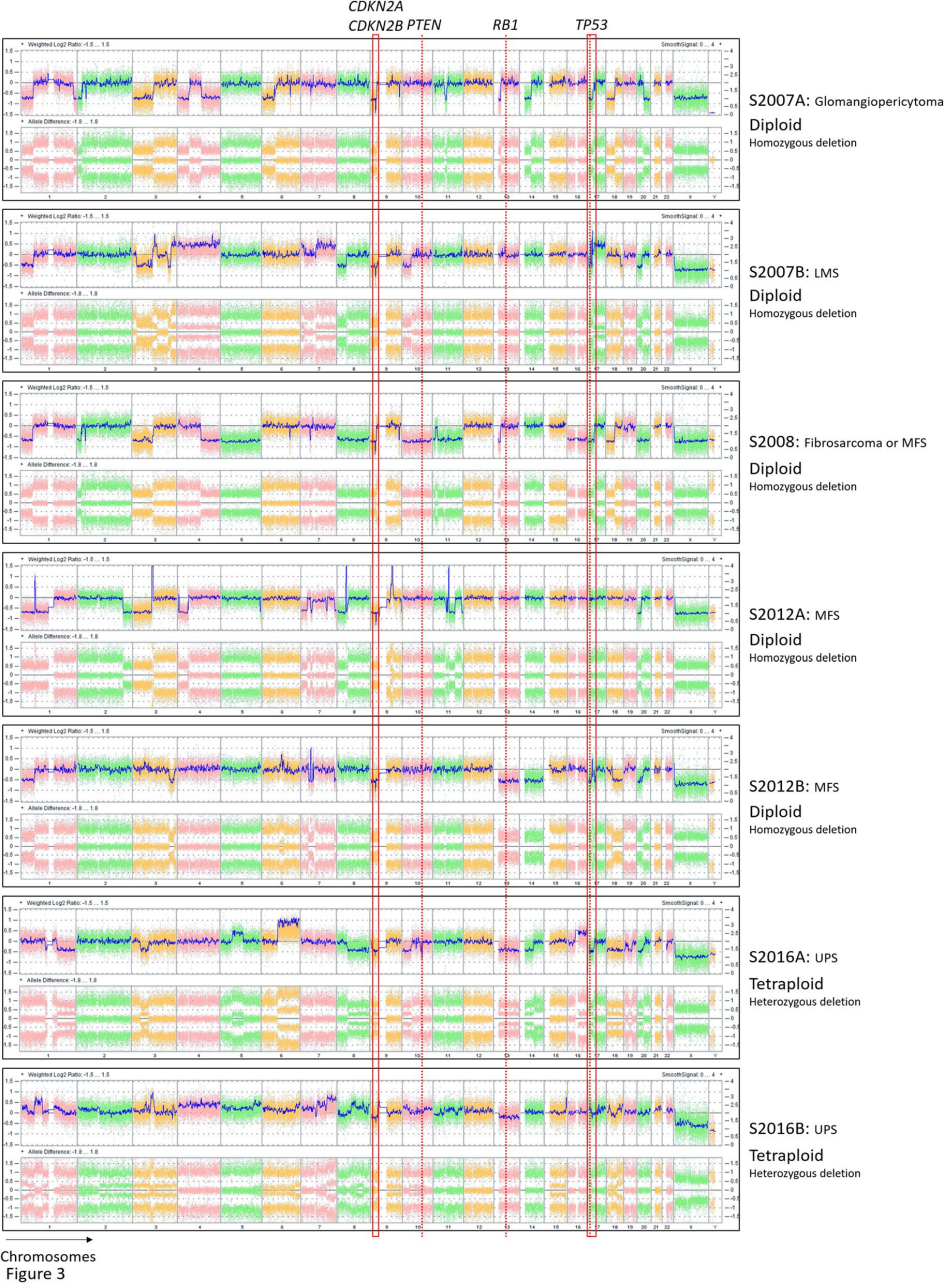
Tumor genomic profiles. Copy number variations (CNVs) and allele frequency differences, plotted on the upper and lower lane of each panel respectively, demonstrate that all tumors present different genomic profiles except for S2007A and S2008 which have alterations in common. Deletion of *CDKN2A* and *CDKN2B* genes on the chromosome 9 is highlited. Ploidie of each tumor is indicated near each profile and the status of the *CDKN2A/CDKN2B* deletion in each tumor is defined. x axis: chromosome 1 to chromosome Y; y axis: weighted log2(ratio) (upper lane) and allele difference (lower lane).

To assess whether the tumors were genetically related to each other, we first characterized the glomangiopericytal tumor and the six sarcomas by DNA array. The seven tumors presented losses and more rarely gains, mainly involving whole chromosomes or chromosome arms (Fig. 3). According to their genomic profiles and allelic status, the tumors developed between 2007 and 2012 had diploid profiles whereas those developed in 2016 were mainly tetraploid. The losses of part of chromosomes 1p, 2p, 8p, 9p, 10p, 17p and 20p were detected in two or more tumors. However, close inspection of the boundaries showed that all alteration breakpoints were different between the tumors, except between S2007A and S2008 (Figure S1). The latter shared several breakpoints (on chromosomes 1p, 2p, 3p 4q, 6q, 7p, 9p, 9q, 13 and 18p), suggesting that S2008 was a local recurrence of S2007A. Genomic profiles of six out of seven tumors were thus very different overall, so they were likely independent.

Following our hypothesis of a constitutional alteration leading to the development of these tumors, we first studied the most frequently altered gene in sarcomas with complex genetics: *TP53*^16^. It showed a heterozygous loss in all tumors except in S2012A and S2016B, again with different breakpoints between the tumors. Regarding other frequently altered genes in sarcomas, one copy of chromosome 13 carrying *RB1* was lost only in S2012B and S2016A and one copy of *PTEN* was lost only in S2008 and S2016A, indicating that these alterations were probably not the primary genetic alterations (Fig. 3).

However, even though the alterations were different between all primary tumors, those affecting the short arm of chromosome 9 have the same consequence in all tumors: the loss of *CDKN2A* and *CDKN2B* genes (Fig. 3). Breakpoints for these losses were different between the tumors, except for S2007A and S2008 (Figure S2A). The upstream breakpoints in S2007A/S2008 and S2012A were located at different positions in the *MLLT3* gene (Figure S2A). According to the weighted Log2ratio and allele difference, the loss of *CDKN2A* and *CDKN2B* was homozygous in the tumors developed between 2007 and 2012 (Fig. 3 and Figures S2A and B). Regarding the tetraploid tumors developed in 2016, the loss was heterozygous with two copies left.

### Constitutional analysis of CDKN2A/CDKN2B

Constitutional *CDKN2A/B* deletions have already been observed in patients with Melanoma-Astrocytoma Syndrome^17^ and in one patient with Li-Fraumeni syndrome^18^. Thus, DNA array was performed in five non-tumor tissues (Table S1). All five genomic profiles showed no such deletion on chromosome 9 nor any copy number variations (Figure S3). Furthermore, both genes were sequenced at the DNA level in non-tumor tissues. No mutation was found in the two genes (data not shown), definitively ruling out any constitutional origin of the *CDKN2A/2B* alteration.

### Fusion transcript and mutation analyses by RNA sequencing

RNA-seq was performed to explore the presence of fusion transcripts as well as the expressed mutational profile in five tumors (S2007A, S2007B, S2008, S2012A, and S2016A).

No fusion transcript shared by all or several tumors was detected, except *EEF1DP3-FRY* which is actually a read-through (Table S5)^19^.

**Figure 4:**
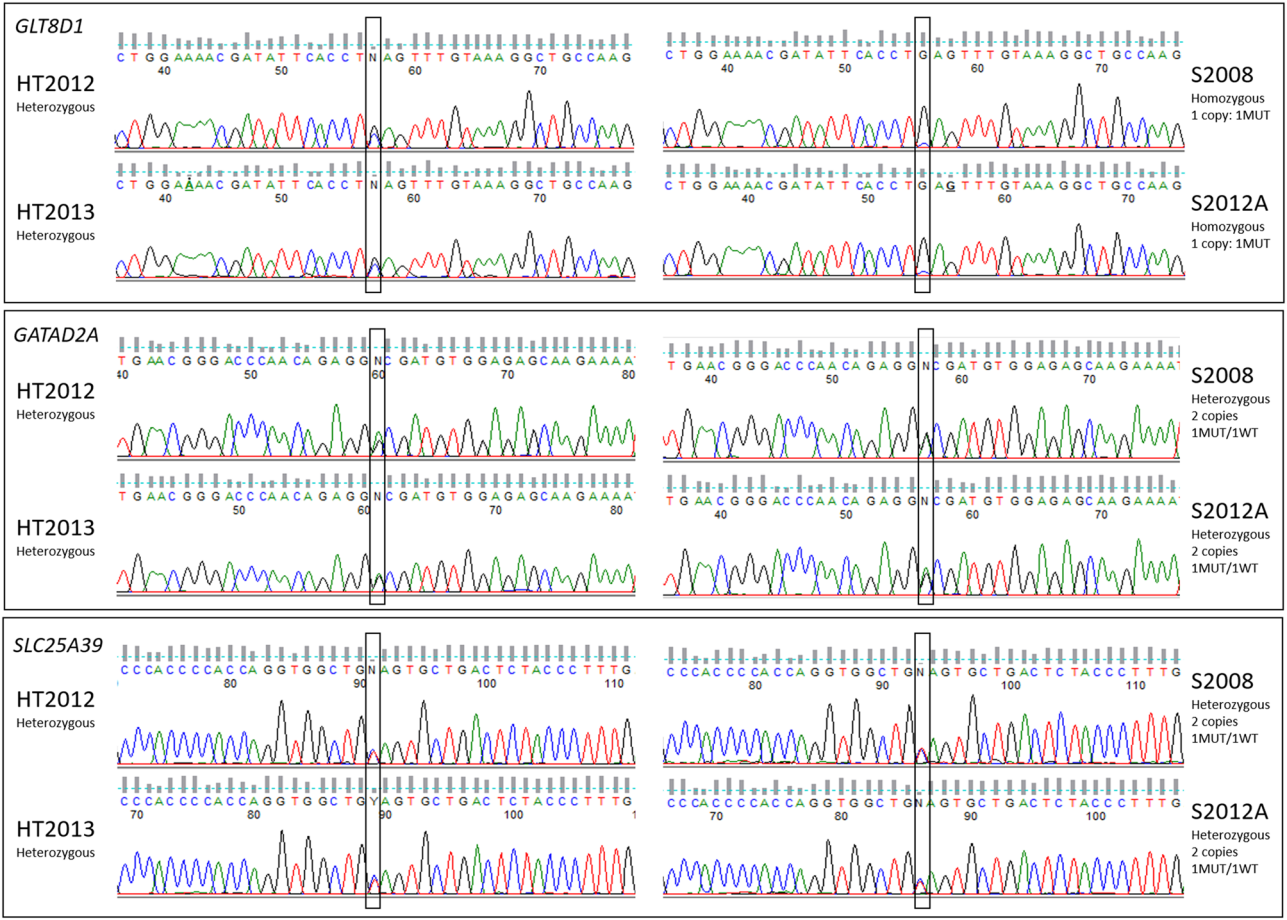
Validated genomic mutations. Sequence chromatograms showing *GLT8D1*, *GATAD2A* and *SLC25A39* mutations observed on genomic DNA in two healthy tissues (HT2012, HT2013) and two tumors (S2008, S2012A) (Sequence viewer: FinchTV, Geospiza). Frames indicate mutation sites (hg38, *GLT8D1*: chr3:g.52695006G > C, *GATAD2A*: chr19:g.19465410A > G, *SLC25A39*: chr17:g.44320429C > T). Allelic status is indicated for each case and number of copies of each gene, determined according to DNA-array data, in each tumor is also presented. *MUT* mutated allele, *WT* wild-type allele.

Regarding mutations, bioinformatic analysis detected 650,497 variations present in at least one tumor. After applying several filters to reduce the number of mutations, only six variations present in all samples were kept as potential real mutations and not polymorphisms or artefacts (Tables S2 and S3). These variations affected the following genes: *AZIN1* (c.A1099G), *COG3* (c.A1903G), *COPA* (c.A490G), *GATAD2A* (c.A65G), *GLT8D1* (c.C955G) and *SLC25A39* (c.C809T)*,* and were all verified by Sanger sequencing on tumors and non-tumor DNA. The variations predicted in *AZIN1*, *COG3* and *COPA* were not found by Sanger sequencing (Table S2), suggesting that they were artefacts. The variations in *GATAD2A* (c.A65G)*,* *GLT8D1* (c.C955G) and *SLC25A39* (c.C809T) were validated in all sequenced cases both in non-tumor and tumor samples (Fig. 4 and Figure S4), showing that these variations were constitutive mutations.

*GATAD2A* and *SLC25A39* mutations were heterozygous in all cases (Fig. 4 and Figure S4) and both alleles were expressed according to RNA-seq data (Table S3). Interestingly, the mutation in *GLT8D1* was associated with a deletion (distinct from one sample to another) of one copy in five of the seven tumors. It was homozygous in four diploid cases (S2007A, S2007B, S2008 and S2012A) and was identified in two copies in the tetraploid S2016A (with one copy of the WT allele) (Fig. 4, Figure S4 and Figure S5). In addition, only the mutated allele was expressed in the five RNA-sequenced cases, and even in S2016A which still retained WT alleles (alternative allele quantification ranges from 82.6% to100% with very low remaining expression of the WT allele likely due to contaminating normal cells) (Table S3).

The three mutations were missense. Substitution in *GATAD2A* (D22G) changed an uncharged polar amino acid (asparagine) to a non-polar amino acid (glycine), while the one in *GLT8D1* (Q319E) led to the replacement of an uncharged polar amino acid (glutamine) by a negatively charged polar amino acid (glutamic acid). Finally, the mutation in *SLC25A39* (A270V) gave rise to the change of an aliphatic hydrophobic non-polar amino acid (alanine) into an amino acid sharing the same features (valine) (Table S3). According to PhosphoSitePlus (v6.5.9.1v6.5.9.1) all three changed amino acids are maintained between human and mouse and are not related to post-translational modifications.

The effect of the missense mutations on the function of the altered gene was therefore unclear. Functional prediction algorithms almost all agreed that the mutation in *GATAD2A* (D22G) was neutral. Predictions about the mutation in *GLT8D1* (Q319E) were more ambiguous with four algorithms in favor of a deleterious effect and five others suggesting that the effect is minor. Finally, nine out of the 10 algorithms used presented the mutation in *SL25A39* (A270V) as deleterious (Table S3). However, regarding the *GLT8D1* mutation, its association with a deletion in five cases and a loss of WT allele expression in all studied tumors suggested a loss of function of the *GLT8D1* gene.

## Discussion

For this unique case of a 72-year-old patient who first presented with glomangiopericytal tumor and then six sarcomas of the extremities between 2007 and 2016, histological examination of the tumors could not discriminate between too clinically distinct situations, i.e. a unique disease with local/distant recurrences or a succession of different diseases.

The first issue was thus to establish whether all the tumors were clonally related or independent from each other. Genomic profile analysis showed that the S2008 tumor was the local recurrence of the S2007A tumor and that the other tumors were not clonally related.

Second, the presence of six different sarcomas in the same patient prompted us to look for a common tumorigenic alteration that might be constitutional. The only shared alteration between all tumors was the loss of the *CDKN2A/2B* genes that resulted from chromosome 9 rearrangements in all non-related tumors, but which is not constitutional because it is not observed in the non-tumor tissues. This alteration has already been described in sarcomas with complex genetics^6,20^. Interestingly, although the breakpoints for these losses are different in each tumor, they all occur in a narrowed region around *CDKN2A/2B* genes, and for three tumors the breaks occurred in the *MLLT3* gene. *MLLT3* is one of the most common fusion partner genes of the *MLL* gene resulting in the t(9;11)(p22;q23) detected in acute myelogenous leukemia (AML) and in acute lymphocytic leukemia (ALL)^21^. Strissel et al*.* identified several common structural DNA elements between *MLLT3* and *MLL* genes and proposed a DNA breakage and repair model in which a non-homologous chromosomal recombination with subsequent DNA repair, could result in translocations between the two genes^21^. Thus, a particular chromatin state of this region may have promoted the rearrangement in *MLLT3* or its surrounding region, leading to the loss of *CDKN2A/2B* loci.

Third, no fusion transcript shared by all tumors was found and only variations in three genes were detected both in tumor and non-tumor tissues: *SLC25A39* (c.C809T)*,* *GLT8D1* (c.C955G) and *GATAD2A* (c.A65G). Analysis in cBioPortal revealed that mutations in these genes have been very rarely reported in sarcomas^22,23^. In the 255 sarcomas of the TCGA PanCancer Atlas Studies, alterations were found for *SLC25A39,* *GLT8D1* and *GATAD2A* in 0.78%, 1.57%, and 2.75% of cases, respectively^24,25^.

*SLC25A39* is located on chromosome 17q21.31 and encodes a protein required for normal heme biosynthesis^26^. Little is known about the functions of SLC25A39 and only its role in erythropoiesis and neural functions have been described^26,27^. The *SLC25A39* missense mutation observed in the patient (A270V) was heterozygous with both alleles expressed and occurred in one of the mitochondrial carrier domains of the protein^28^, potentially modifying the function of the protein.

*GATAD2A* (GATA Zinc Finger Domain Containing 2A), located on chromosome 19p13.11, codes for the p66α protein, which is a subunit of the nucleosome remodeling and histone deacetylation (NuRD) complex, itself implicated in transcription regulation through chromatin compaction and decompaction^29^. At the transcriptional level, the NuRD complex is recruited by tissue-specific oncogenic transcription factors to repress the expression of tumor suppressor genes, while at the post-translational level, it has been shown to deacetylate p53 to inactivate p53-induced apoptosis^30^. It has also been detected at replication forks and ensures proper DNA replication, cellular proliferation and protection of genome integrity^31^. Moreover, it has been shown that GATAD2A/NuRD can be recruited to sites of DNA damage to promote repair by homologous recombination^32^. The mutation found here (D22G) occurred at an amino acid not located in an identified functional domain^33^. Functional prediction algorithms almost all agree that the mutation in *GATAD2A* is neutral. While the NuRD complex is known to play several important emerging roles in cancer biology^31^, the involvement of GATAD2A in cancer is still poorly understood. Nevertheless, a genome-wide meta-analysis of breast, ovarian and prostate cancers identified three cancer susceptibility loci associated with intronic variants of *GATAD2A*^30^.

*GLT8D1* (glycosyltransferase 8 domain containing 1), located on chromosome 3p21.1, encodes a glycosyltransferase enzyme of unknown function, ubiquitously expressed and localized in the Golgi apparatus^34^. Amyotrophic Lateral Sclerosis (ALS), a severe neurodegenerative disorder, has been ascribed to missense mutations in *GLT8D1*^35^. All mutations found in familial ALS cases and in early-onset sporadic ALS arise in *GLT8D1* exon 4, which encodes the substrate-binding domain of GLT8D1 and is associated with reduced enzymatic activity. Thus, pathogenic *GLT8D1* mutations are thought to be autosomal dominant mutations that are associated with haploinsufficiency and/or a dominant-negative effect^34^. The missense *GLT8D1* mutation found in the patient (Q319E) is located in exon 10 and the modified amino acid is located in the glycosyltransferase domain of the protein, like ALS mutations, but in a more C-terminal part^35^. GLT8D1 overexpression was also recently reported in melanoma to be associated with worse overall survival and progression-free survival^36^. Unfortunately, no material was available from the melanoma in our patient. Consensus is lacking about the deleterious effect of this mutation. Deletion of *GLT8D1* in five tumors and homozygous expression of the mutated allele in all of them, is consistent with a loss of function of the *GLT8D1* gene. The deletion could thus be an early event acquired during the first steps of the oncogenic process, suggesting that its functional loss participates in the tumor inception.

Finally, a potential role for *SLC25A39* and *GATAD2A* in tumor development in this patient is weakly supported by literature and the most likely candidate gene appears to be *GLT8D1.*

The predisposing impact of this mutation must be moderate because, even if the patient developed many tumors, they all occurred late in life. It’s more likely that it could deregulate aging cells. In addition, the cell type in which this deregulation occurred might be at the origin of the different types of cancer developed by the patient. For example, if melanocytes were to be affected, this would result in a melanoma^37^. Likewise, if different mesenchymal cell types are impacted, it could lead to the development of LMS, UPS or MFS^38,39^.

Obviously, a thorough experimental evaluation is mandatory to conclude on the definitive involvement of one of these candidates in sarcoma’s, predisposition/inception.

## Supplementary Information

Supplementary Figure S1.

Supplementary Figure S2.

Supplementary Figure S3.

Supplementary Figure S4.

Supplementary Figure S5.

Supplementary Table S1.

Supplementary Table S2.

Supplementary Table S3.

Supplementary Table S4.

Supplementary Table S5.
