# Supplementary material for: Germinal GLT8D1, GATAD2A and SLC25A39 mutations in a patient with a glomangiopericytal tumor and five different sarcomas over a 10-year period
Source: Sci Rep. 2021 May 7;11:9765. doi: 10.1038/s41598-021-88671-0 (PMC8105326; doi:10.1038/s41598-021-88671-0)
Supplement: Supplementary file 1 — Supplementary Figure S1. [file 41598_2021_88671_MOESM1_ESM.pdf]

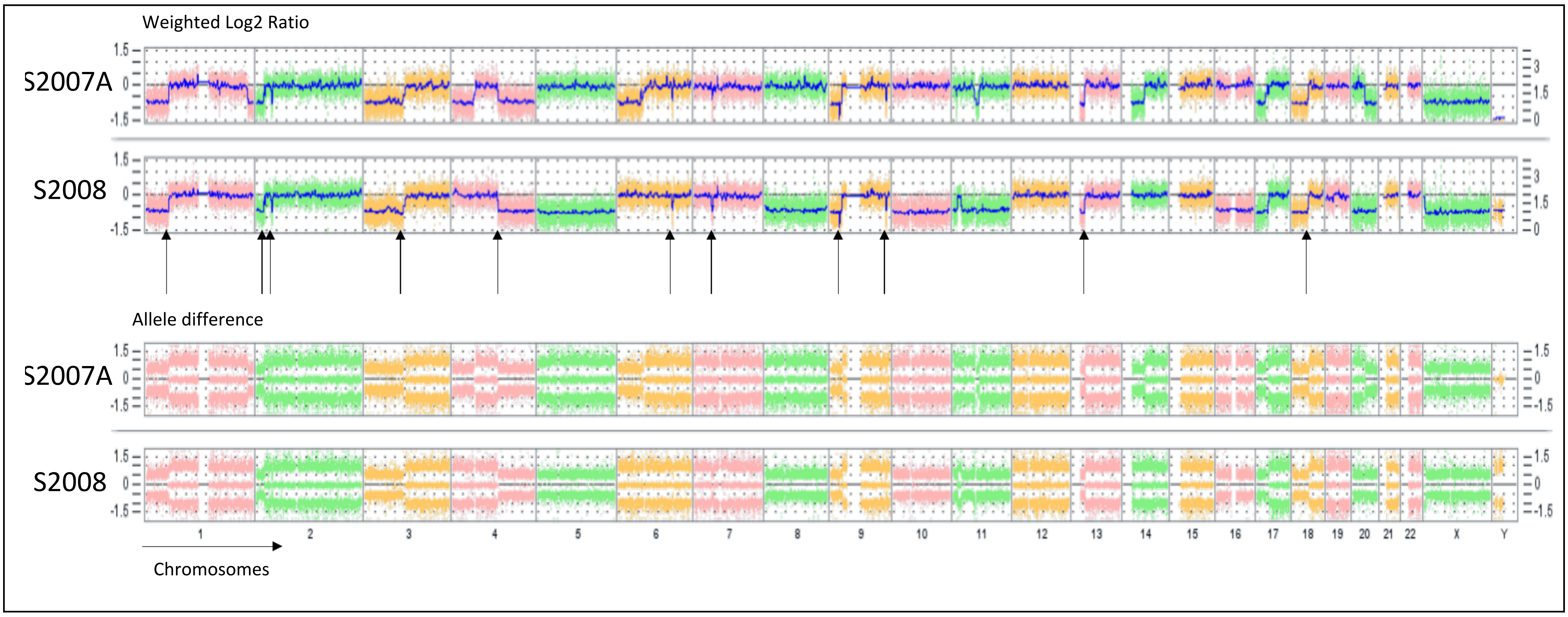

Figure S1. Comparison of S2007A and S2008 genomic profiles.

Comparisons of copy number variation (CNV) profiles of both tumors on the upper lane and allele frequency differences on the lower lane, show that S2007A and S2008 have many alterations in common with identical breakpoints indicated by arrows on the CNV profile. x axis: chromosome 1 to chromosome Y; y axis: weighted log2(ratio) (upper lane) and allele difference (lower lane).
