# Supplementary material for: Germinal GLT8D1, GATAD2A and SLC25A39 mutations in a patient with a glomangiopericytal tumor and five different sarcomas over a 10-year period
Source: Sci Rep. 2021 May 7;11:9765. doi: 10.1038/s41598-021-88671-0 (PMC8105326; doi:10.1038/s41598-021-88671-0)
Supplement: Supplementary file 2 — Supplementary Figure S2. [file 41598_2021_88671_MOESM2_ESM.pdf]

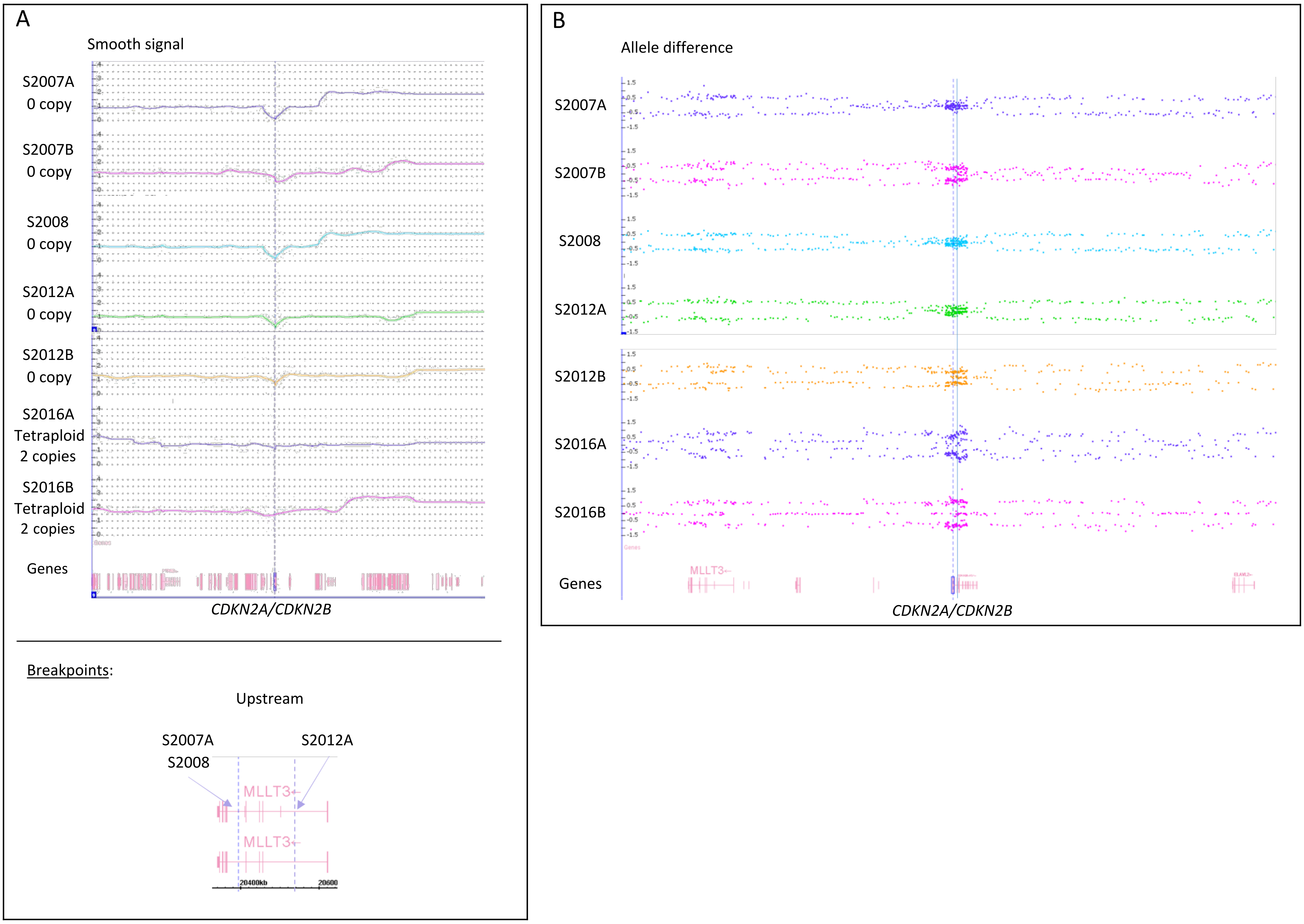

Figure S2. *CDKN2A* and *CDKN2B* deletion in tumors.

A: Smooth signal of part of the chromosome 9 short arm is presented for each of the seven tumors. *CDKN2A/CDKN2B* gene location is indicated by a dotted line. Number of *CDKN2A/CDKN2B* copies is given for each case and ploidy for multiploid cases is indicated. Upstream breakpoints in *MLLT3* gene are presented for S2007A/S2008 and S2012A. **B**: Allelic status of part of chromosome 9 short arm for each tumor confirms a homozygous deletion of the genes for the five first tumors with a slight contamination with normal cells in S2007B and S2012B. Regarding S2016A and S2016B, two copies of *CDKN2A* and *CDKN2B* genes are retained.
