# Supplementary material for: Germinal GLT8D1, GATAD2A and SLC25A39 mutations in a patient with a glomangiopericytal tumor and five different sarcomas over a 10-year period
Source: Sci Rep. 2021 May 7;11:9765. doi: 10.1038/s41598-021-88671-0 (PMC8105326; doi:10.1038/s41598-021-88671-0)
Supplement: Supplementary file 3 — Supplementary Figure S3. [file 41598_2021_88671_MOESM3_ESM.pdf]

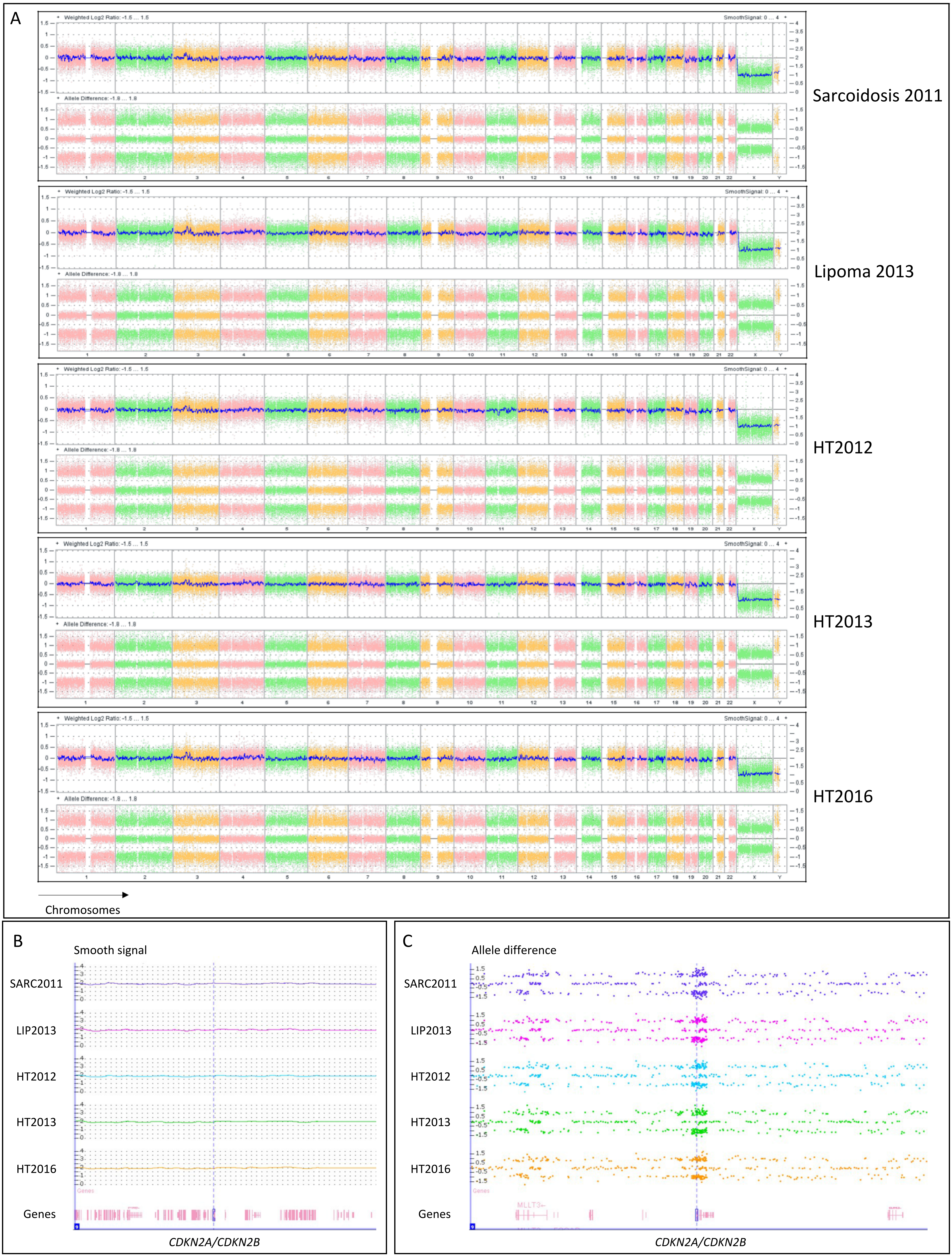

A
Sarcoidosis 2011
Lipoma 2013
HT2012
HT2013
HT2016
Weighted Log2 Ratio: -1.5 ... 1.5
SmoothSignal: 0 ... 4
Allele Difference: -1.8 ... 1.8
Chromosomes
B
Smooth signal
SARC2011
LIP2013
HT2012
HT2013
HT2016
Genes
CDKN2A/CDKN2B
C
Allele difference
SARC2011
LIP2013
HT2012
HT2013
HT2016
Genes
MLLT3
CDKN2A/CDKN2B

Figure S3. Malignancies and healthy tissue genomic profiles.

A: Copy number variations (CNVs) and allele frequency differences, plotted on the upper and lower lane of each panel respectively, show that sarcoidosis tissue, lipoma and the three healthy tissues present no CNV. x axis: chromosome 1 to chromosome Y; y axis: weighted log2(ratio) (upper lane) and allele difference (lower lane). **B**: Smooth signal of part of chromosome 9 short arm is presented for each of the five tissues. *CDKN2A/CDKN2B* gene location is indicated by a dotted line. No *CDKN2A* and *CDKN2B* gene deletion could be observed on chromosome 9. **C**: Allelic status of part of chromosome 9 short arm for each tissue confirms that there is no *CDKN2A* and *CDKN2B* gene deletion in these samples.
