# Supplementary material for: Germinal GLT8D1, GATAD2A and SLC25A39 mutations in a patient with a glomangiopericytal tumor and five different sarcomas over a 10-year period
Source: Sci Rep. 2021 May 7;11:9765. doi: 10.1038/s41598-021-88671-0 (PMC8105326; doi:10.1038/s41598-021-88671-0)
Supplement: Supplementary file 4 — Supplementary Figure S4. [file 41598_2021_88671_MOESM4_ESM.pdf]

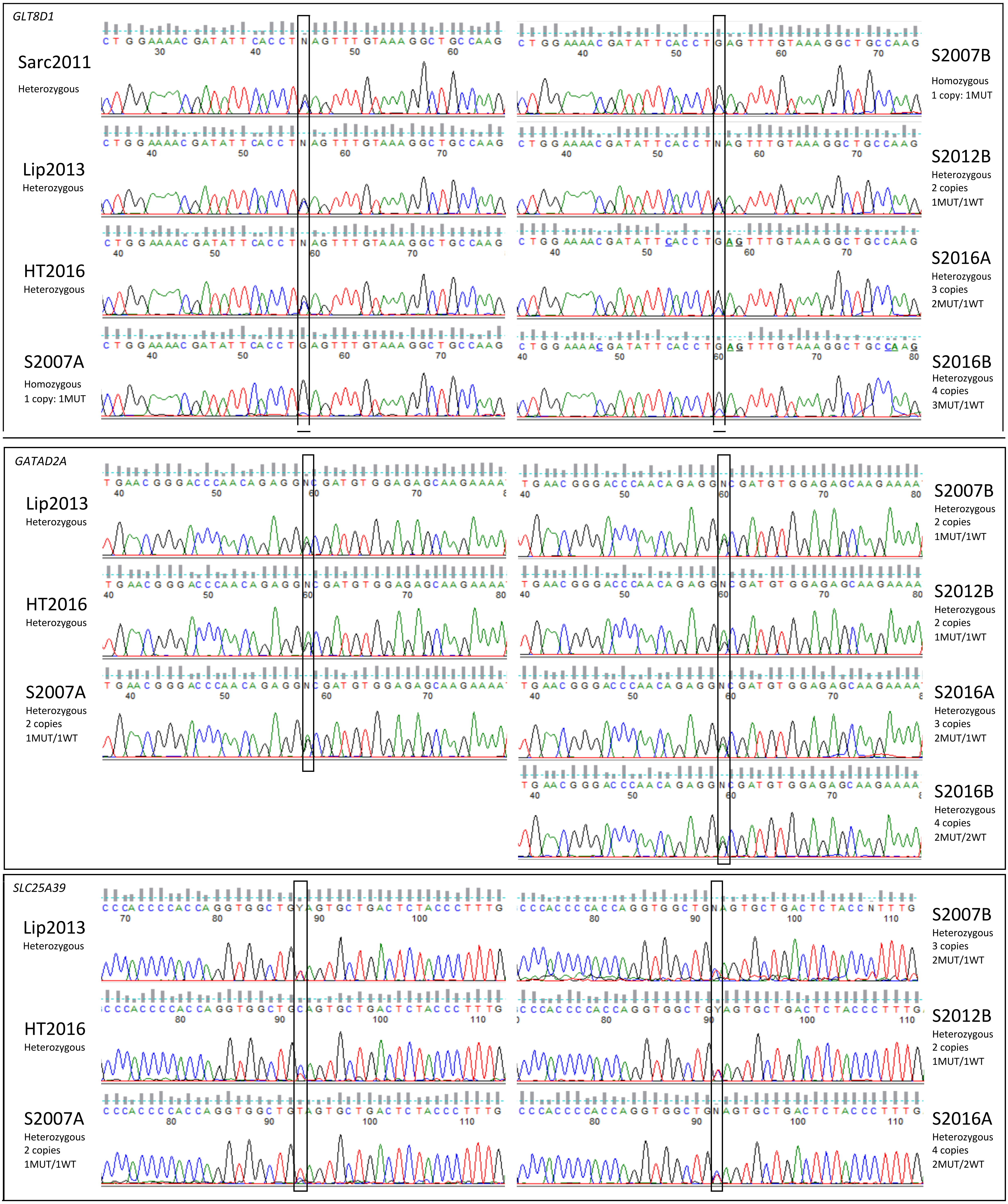
GLT8D1
Sarc2011
Heterozygous
Lip2013
Heterozygous
HT2016
Heterozygous
S2007A
Homozygous
1 copy: 1MUT
S2007B
Homozygous
1 copy: 1MUT
S2012B
Heterozygous
2 copies
1MUT/1WT
S2016A
Heterozygous
3 copies
2MUT/1WT
S2016B
Heterozygous
4 copies
3MUT/1WT
GATAD2A
Lip2013
Heterozygous
HT2016
Heterozygous
S2007A
Heterozygous
2 copies
1MUT/1WT
S2007B
Heterozygous
2 copies
1MUT/1WT
S2012B
Heterozygous
2 copies
1MUT/1WT
S2016A
Heterozygous
3 copies
2MUT/1WT
S2016B
Heterozygous
4 copies
2MUT/2WT
SLC25A39
Lip2013
Heterozygous
HT2016
Heterozygous
S2007A
Heterozygous
2 copies
1MUT/1WT
S2007B
Heterozygous
3 copies
2MUT/1WT
S2012B
Heterozygous
2 copies
1MUT/1WT
S2016A
Heterozygous
4 copies
2MUT/2WT

Figure S4. Validated genomic mutations.

Sequence chromatograms showing *GLT8D1*, *GATAD2A* and *SLC25A39* mutations observed on genomic DNA in malignancies, healthy tissues and tumors not presented in Figure 4. Frames indicate mutation sites (*GLT8D1*: NM_018446:c.955C>G, *GATAD2A*: NM_017660:c.65A>G, *SLC25A39*: NM_001143780:c.809C>T). Allelic status is indicated for each case and number of copies of each gene, determined according to DNA-array data, in each tumor is also presented. HT: healthy tissue, S: sarcoma, Sarc: sarcoidosis, Lip: lipoma, MUT: mutated allele, WT: wild-type allele.
