# Supplementary material for: Germinal GLT8D1, GATAD2A and SLC25A39 mutations in a patient with a glomangiopericytal tumor and five different sarcomas over a 10-year period
Source: Sci Rep. 2021 May 7;11:9765. doi: 10.1038/s41598-021-88671-0 (PMC8105326; doi:10.1038/s41598-021-88671-0)
Supplement: Supplementary file 5 — Supplementary Figure S5. [file 41598_2021_88671_MOESM5_ESM.pdf]

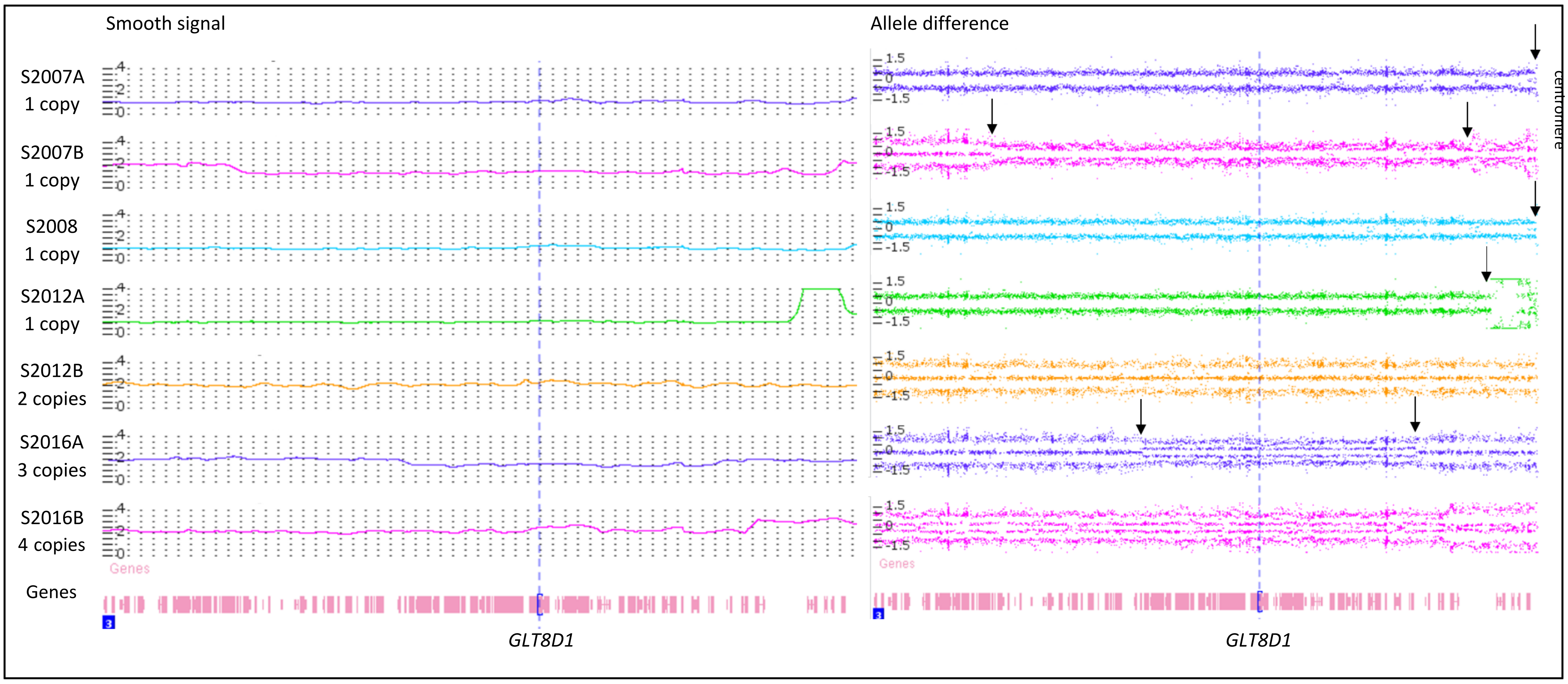

Figure S5. *GLT8D1* genomic status.

Smooth signal and allele difference corresponding to chromosome 3 short arm are presented for each of the seven tumors. *GLT8D1* gene location is indicated by a dotted line and breakpoints regarding the deletion on chromosome 3 in five tumors are indicated by arrows on the allele difference profiles. Number of *GLT8D1* copies is given for each case.
